# Grape seed extract and L-ascorbic acid exert antineoplastic effects against solid Ehrlich carcinoma *in vivo* by modulating the tumor microenvironment and Th1/Th2 balance

**DOI:** 10.3389/fimmu.2025.1635071

**Published:** 2025-08-06

**Authors:** Dalia S. Morsi, Heba M. R. Hathout, Hind S. AboShabaan, Mahmoud Emam, Manal El-khadragy, Ahmed E. Abdel Moneim, Islam M. El-Garawani, Hagar A. Abu Quora

**Affiliations:** ^1^ Zoology Department, Faculty of Science, Menoufia University, Shibin El Kom, Egypt; ^2^ Natural Resources Department, Faculty of African Postgraduate Studies, Cairo University, Giza, Egypt; ^3^ Clinical Pathology Department, National Liver Institute Hospital, Menoufia University, Shibin El Kom, Egypt; ^4^ Phytochemistry and Plant Systematics Department, National Research Centre, Giza, Egypt; ^5^ Department of Biology, College of Science, Princess Nourah bint Abdulrahman University, Riyadh, Saudi Arabia; ^6^ Zoology and Entomology Department, Faculty of Science, Helwan University, Cairo, Egypt; ^7^ Al-Ayen Scientific Research Center, Al-Ayen Iraqi University, AUIQ, An Nasiriyah, ThiQar, Iraq

**Keywords:** grape seed, vitamin C, solid ehrlich carcinoma, tumor immune microenvironment, Th/Th2 balance

## Abstract

**Objective:**

The existing study sought to highlight the modulatory effect of co-treatment based on grape seed extract (GSE) and L. ascorbic acid (AA) on tumor microenvironment and immune response in murine solid Ehrlich carcinoma (SEC).

**Methods:**

GSE (200 mg / kg; orally) and AA (50 mg/ kg; orally) were given either separately or in a combination for 14 days. GSE active metabolites were identified using GC-MS and LC-MS/MS. Tumor size, Ki-67, Caspase-3, intratumoral infiltrated CD4+, CD8+ and FOXP3+ cells were detected immunohistochemically. Oxidative stress of tumor cells was determined. Serum levels of IL-12, IFN-γ, IL-4 and IL-10 were detected using ELISA.

**Results and discussion:**

The results revealed treatment with GSE and/or AA markedly diminished tumor size, intensified intratumoral oxidative stress, downregulated tumor cell proliferation along with upregulated tumor cells’ apoptosis. GSE and AA enhanced tumor immune microenvironment through increasing CD8+ and CD4+ T cells accompanied by decreasing FOXP3+ Treg cells infiltrated in tumors. GSE and/ or AA moved Th1/Th2 balance in favor of Th1 as evidenced by increased serum levels of IFN-γ and IL-12 accompanied with decreased serum levels of IL-4 and IL-10. These findings may be attributed to the presence of different chemical scaffolds of phenolic acids, Flavan-3-ols and its glycosides, glycerolipids and its glycosides, glycosylated seco-iridoids, dihydrochalcone, stilbenoid, flavone, dihydroxyflavone, and methylated flavone, sugars, and fatty acids. In conclusion, results suggested that dual treatment based on GSE & AA are promising anticancer therapeutics, through their potency to control proliferation, induce apoptosis, intratumoral oxidative stress, modulate tumor immune microenvironment and shifting Th1/Th2 response toward Th1

## 1 Introduction

Cancer is considered one of the leading causes of death across various communities (1). In the USA alone, it is projected that there will be approximately 2,041,910 new cancer cases and 618,120 cancer-related deaths in 2025 (2). For decades, radiotherapy, chemotherapy, and surgery have remained the standard treatment modalities for cancer (3). However, the effectiveness of chemotherapeutic agents is limited by several factors, including the emergence of drug-resistant cancer cells, low drug sensitivity, and severe side effects (4, 5). These challenges highlight the need to develop novel supplemental or alternative therapeutic strategies to eradicate tumor cells (6, 7).

New anticancer entities are often found in natural materials. Recently, more than 60% of commercial drugs have been derived from natural sources, including bacteria, fungi, plants, and animals (4, 8–11). Food offers additional bioactive substances for promoting health and preventing disease, in addition to the vital nutrients required for life. Consumption of grains, fruits, and vegetables has been strongly linked to a lower risk of numerous serious illnesses, such as cancer (12–14). Combination treatment has shown the best outcomes regarding anticancer properties; its superiority lies in its capacity to target multiple pathways, which effectively decreases drug resistance, as cancer cells often cannot adjust to the concurrent harmful effects of two therapeutic drugs (15). By using treatment combinations to target different pathways, the likelihood of disease control can be increased, and the likelihood of cancer cells becoming more aggressive and incurable reduced. In many instances, combination treatment can also lower the dose requirements for each medication, which lessens adverse effects when compared to monotherapy, although some treatment combinations have been demonstrated to enhance toxicity (16, 17). Combination therapy also has the benefit of targeting all cancer cells that might contribute to drug resistance and cancer recurrence after remission in later years (18, 19).

The grape (*Vitis vinifera* L.) is one of the most well-known fruit crops worldwide (20), and it has long been valued as a traditional remedy with important uses in curing a range of human ailments. Grapes are among the most commonly cultivated fruit crops and are considered to be the most consumed fruit globally; they can be found growing in many regions (21). Grape seeds, a byproduct of the wine and juice industry, are an excellent source of biologically active substances (22). Approximately, seeds account for 5% of the whole grape weight, representing 40%–50% of the solid waste in grape industries. Grape seeds contain about 60%–70% of the polyphenols, compared to 28%–35% in peels and 10% in fruits (22–24). Antioxidants found in high concentrations in grape seeds, such as phenolic compounds, can reduce the risk of chronic illness by preventing damage caused by free radicals. Epicatechin, epicatechin-3-*O*-gallate, and catechin, often known as proanthocyanidins (PAs), are abundant in grape seeds. PAs found in grape seeds have anti-inflammatory, antiallergic, and antiarthritic qualities. They also scavenge free oxygen radicals, prevent skin aging, and reduce peroxidation activity linked to UV radiation (25, 26). Improved antioxidant, cardiovascular, anti-inflammatory, antiviral, and anticancer properties have all been attributed to polyphenols (27–29).

Ascorbic acid (vitamin C) is a fundamental micronutrient that humans cannot synthesize on their own due to mutations in the gene that encodes the final enzyme in its biosynthesis route (30, 31). As an essential component of enzymes involved in processes and outcomes critical to cancer development, vitamin C contributes to a variety of processes, including antioxidant defense, transcription, and epigenetic regulation of gene expression (32). Vitamin C has also been found to positively affect the immune system and inflammatory responses, which is important for the host’s ability to fight off precancerous and cancerous cells (33). The over-the-counter medication vitamin C is heavily promoted as a health supplement. It is accessible to the general population in dosages higher than the current Dietary Reference Intake, which is 75 mg for women and 90 mg for men daily. Although there has long been no solid medical evidence supporting high dosages, they have been employed to achieve certain therapeutic outcomes (34).

In this context, this research was performed to explore the potential antitumor and immunomodulatory roles of dual treatment based on red grape seed extract plus vitamin C in a solid Ehrlich carcinoma murine model. To the best of our knowledge, no previous study has addressed the efficacy of dual treatment based on grape seed extract and vitamin C, considering the tumor immune microenvironment and Th1/Th2 response.

## 2 Materials and methods

### 2.1 Plant materials and extract production

Grapes were obtained from the local market and identified and authenticated by a professor of Plant Taxonomy, Botany Department, Faculty of Science, Menoufia University. Grape seeds (*Vitis vinifera* L.) were isolated, left to dry, and ground into a fine powder. A total of 37 g of the powder was macerated and extracted using water. The resulting solution was filtered and evaporated using Rotavapor^®^ (Heidolph, Germany), yielding 2.4763 g of dry extract (~ 6.69%).

### 2.2 Phytochemical studies

#### 2.2.1 Total phenolic and flavonoid content

**Figure 1:**
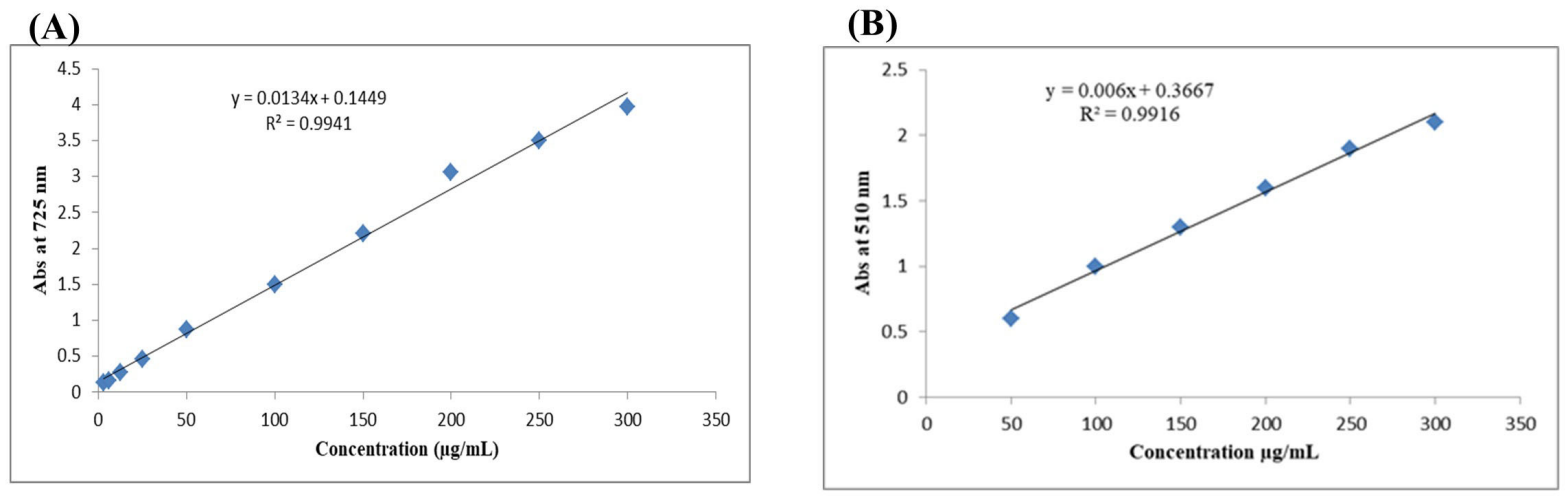
Calibration curves for total phenolic content (TPC) using gallic acid **(A)** and total flavonoid content (TFC) using rutin **(B)**.

The content of phenolic compounds was estimated using gallic acid as a standard at concentrations of 3.125–300 μg mL^−1^ to construct a calibration curve, with an average *R* ^2^ = 0.9941, as shown in **Figure 1A**. The method employed was comparable to that described by Zhang et al. (35). In addition, rutin was used as a standard at concentrations of 50–300 μg mL^−1^ to construct a calibration curve for determining flavonoid content, with average *R* ^2^ = 0.9916 (**Figure 1B**) (36).

#### 2.2.2 Gas chromatography-mass spectrometry sample derivatization and analysis

Before application to gas chromatography-mass spectrometry (GC-MS), the grape seed extract (GSE) sample was extracted, prepared, and treated with a silylation reagent to derivatize the functional groups into trimethylsilyl (TMS) derivatives (37). A 1-µL aliquot of the sample was then injected into the GC-MS system (Agilent Technologies; GC: 7890B with MS detector; 5977A) at the Central Laboratories Network, National Research Centre, Cairo, Egypt, funded through project number 13060131. The column specifications, carrier gas, flow rate, mass ionization energy (70 eV), temperature, and spectral range were previously described in detail. Constituents were identified by comparing the fragmentation patterns with data from the Wiley and NIST Mass Spectral Libraries.

#### 2.2.3 LC/MS/MS conditions and parameters (instrument, ionization mode)

The GSE was dissolved and analyzed using liquid chromatography–electrospray ionization–tandem mass spectrometry (LC-ESI-MS/MS, SCIEX Triple Quad 5500+ MS/MS). The separation column type, mobile phase gradient, flow rate, ionization mode (negative), ion spray voltage, source temperature, collision energy, and ion source settings were previously described (38, 39).

### 2.3 Animals

Female Swiss CD1 mice (*Mus musculus*), aged 6–8 weeks (weighing 26–30 g), were obtained from the National Research Center in Giza, Egypt. All mice were housed under controlled laboratory conditions with a 12-h light/dark cycle. Standard rodent chow and water were made available *ad libitum*. Prior to the start of the experiments, mice were acclimated to the laboratory environment for 12 days. All animal handling procedures were conducted in accordance with the guidelines of the Institutional Animal Care and Use Committee (IACUC) of Menoufia University, Egypt. The study protocol was approved by the IACUC ethics review board, Faculty of Science (ID: MUFS/F/IM/3/22).

### 2.4 Reagents and antibodies

Doxorubicin, isoflurane, and l-ascorbic acid were obtained from Sigma (St Louis, MO, USA). ELISA kits for interleukin (IL)-10, IL-4, IL-12, and interferon gamma (IFN-γ) were purchased from CUSABIO (Houston, TX, USA). Monoclonal anti-Ki-67 (Abcam (Cambridge, UK) Cat. No. ab16667, RRID: AB_302459, clone: SP6) and anticaspase-3 (Abcam Cat. No. ab184787, RRID: AB_2827742, clone: EPR18297), antimouse CD4 mAb (BD Biosciences (USA) Cat. No. 553729, RRID: AB_395013, clone: GK1.5), antimouse CD8 mAb (BD Biosciences Cat. No. 550372, RRID: AB_393643), and antimouse FOXP3 mAb (Abcam Cat. No. ab22510, RRID: AB_447114). All other compounds used in this study were of the highest grade.

### 2.5 Tumor challenge and treatment protocol

Ehrlich ascites carcinoma (EAC) cells were obtained from the National Cancer Institute’s Research Unit at Cairo University, Egypt. The cells were maintained alive by repeated intraperitoneal implantation of 0.5 × 10^6^ viable tumor cells suspended in 0.2 mL of saline into female Swiss CD1 mice. EAC cells were authenticated based on morphological characteristics, and cell viability was assessed using the trypan blue exclusion assay prior to inoculation. Solid Ehrlich carcinoma (SEC) was induced on day 0 by intramuscular (i.m.) injection of EAC cells (2.5 × l0^6^) into the right thigh of the lower limb of each mouse (7). Cell transfers were conducted under fully sterile conditions. Seventy-two apparently healthy female Swiss CD1 mice were randomly divided into eight groups, each containing nine mice, and categorized as follows:

Group I (Naïve): Mice were not given any treatment or tumors.
Group II (AA): Nontumorized mice received 50 mg/kg l-ascorbic acid (40) by oral gavage for 14 successive days.
Group III (GSE): Nontumorized mice were given 200 mg/kg GSE (41) by oral gavage for 14 consecutive days.
Group IV (SEC): SEC-bearing nontreated mice were inoculated i.m. with 2.5 × l0^6^ EAC cells on day 0, as previously mentioned.
Group V (DOX): SEC-bearing mice were injected i.p. with doxorubicin (4 mg/kg b.wt) every 4 days (10th, 14th, 18th, and 22nd days), according to Khedr and Khalil (42).
Group VI (SEC + AA): SEC-bearing mice were administered AA by oral gavage (50 mg/kg b.wt) for 14 successive days from the 10th day till the 24th day as the same as in Group II.
Group VII (SEC +GSE): SEC-bearing mice were administered GSE by oral gavage (200 mg/kg b.wt) for 16 successive days from the 10th day until the 24th day as the same as in Group III.
Group VIII (SEC+GSE+AA): SEC-bearing mice were co-administered with AA (50 mg/kg bwt, orally) and GSE (200 mg/kg b.wt, orally) for 14 successive days from the 10th day till the 24th day as the same as in Groups II and III.

Mice in good general health without prior illness that developed a measurable solid tumor following SEC inoculation were included in the subsequent investigations. Mice that did not develop a measurable solid Ehrlich tumor after cell injection were excluded. Random assignment to treatment groups was performed to ensure unbiased group allocation. To minimize experimental bias, blinding was implemented during data collection and analysis. Investigators responsible for measuring tumor volumes, conducting immunological assays, and performing histopathological evaluations were blinded to the treatment groups. Group identities were revealed only after the completion of all experimental procedures and data analyses.

### 2.6 Tumor size assessment

A two-end electronic digital caliper (Switzerland) was used to measure the dimensions of the right thigh twice weekly, starting 7 days after tumor induction. Tumor size changes were calculated using the following formula, as described by Goto et al. (43):

Tumor size (mm^3^) = (Tumor’s higher diameter × Tumor’s lower diameter^2^)/2.

Furthermore, tumor volume reduction rate(TVRR%) was computed using the formula: TVRR% = the tumor volume reduction rate [(tumor volume in control − tumor volume in treated group)/tumor volume in control group) × 100] (44).

### 2.7 Sample collection

On day 25, mice were anesthetized with isoflurane, and blood was collected via orbital bleeding. Tumors were excised and divided into two portions. One portion was dissected, weighed, and homogenized in 0.9% normal saline for antioxidant and oxidative stress biomarker determination. The other portion was fixed in 10% neutral formaldehyde for histological and immunohistochemical analyses.

### 2.8 Oxidative stress and antioxidant markers

Tumor tissue homogenates were used to assess oxidative stress and antioxidant biomarkers. Superoxide dismutase (SOD) activity was determined by measuring superoxide anion levels via nitroblue tetrazoluim formazan color development, according to Masayasu and Hiroshi (45). One unit of SOD activity (defined as the enzyme concentration causing 50% inhibition) corresponds to 7.47 g mL^−1^ in the reaction mixture. Reduced glutathione (GSH) levels in tumor tissue were measured following the method of Beutler et al. (46). Catalase (CAT) was determined according to Sinha (47). Lipid peroxidation (LPO) content was assessed by measuring thiobarbituric acid-reactive substances, following the method of Yang et al. (48). To quantify the concentration of nitric oxide (NO), the Griess reagent was utilized, as described by Green et al. (49).

### 2.9 Detection of tumor cell apoptosis, proliferation, and infiltrated immune cells

Tumor tissues fixed in 10% neutral formalin were processed and immunostained for nuclear proliferating protein Ki-67, caspase-3, CD4, CD8, and FOXP3 mAbs, following the manufacturer’s instructions. Immunohistochemical (IHC) staining was performed according to Arriazu et al. (50). Digital IHC images were analyzed using a semiquantitative counting method (Fiji-Image J software, Java-based application for image analysis) (http://imagej.nih.gov/ij/). Six randomly selected high-power fields (× 40) were evaluated to calculate the area fraction, representing the proportion of the immunoreactive area (51).

### 2.10 Cytokine serum levels

Blood samples were centrifuged at 3,000 × *g* for 15 min at 4°C. Serum was then isolated and stored at − 80°C. Cytokine levels of IL-4, IL-10, IL-12, and IFN-γ were detected using the ELISA method. Optical density was recorded at a wavelength of 450 nm, and cytokine concentrations were calculated by comparing the optical density to a standard curve.

### 2.11 Statistical analysis

One-way analysis of variance (ANOVA) followed by Tukey’s *post-hoc* testing, and two-way ANOVA for tumor size, were used to evaluate the results. When ANOVA assumptions were violated, the Kruskal–Wallis test was applied, with *p*-values < 0.05 considered statistically significant. Data were plotted using GraphPad Prism version 8.4 for Windows (GraphPad Software, San Diego, CA, USA).

## 3 Results

### 3.1 Phytochemical results

**Figure 2:**
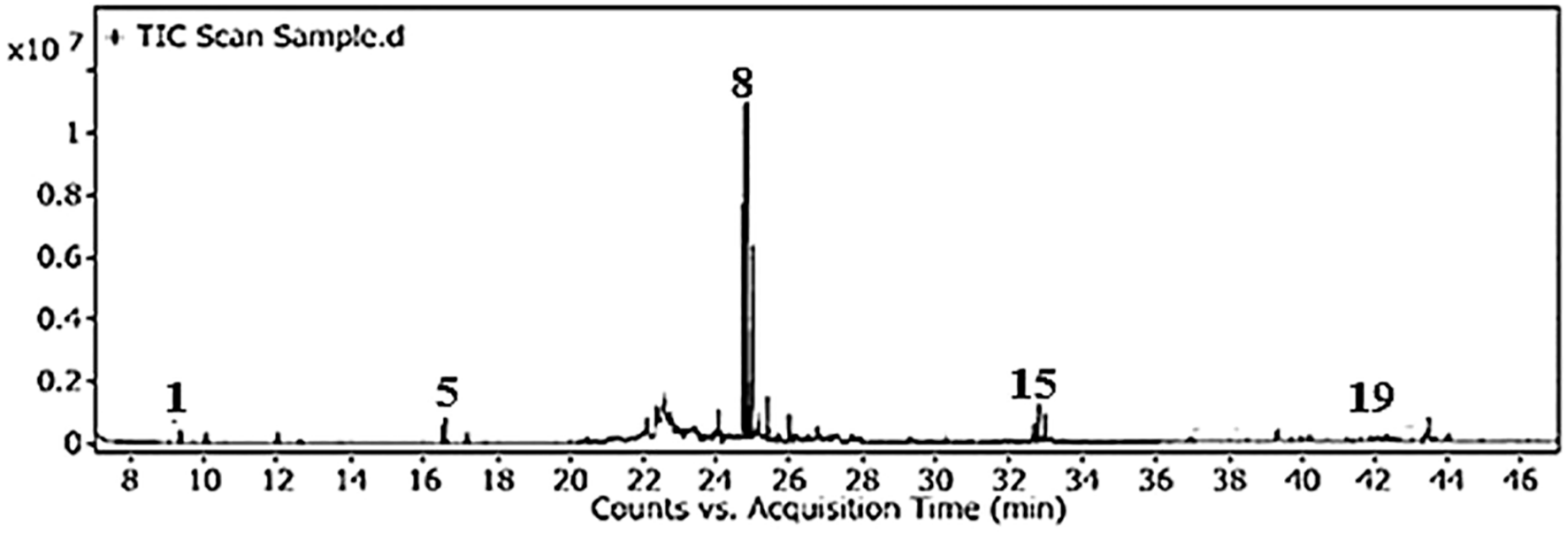
Total ion chromatogram of GSE obtained by GC-MS after silylation.

**Figure 3:**
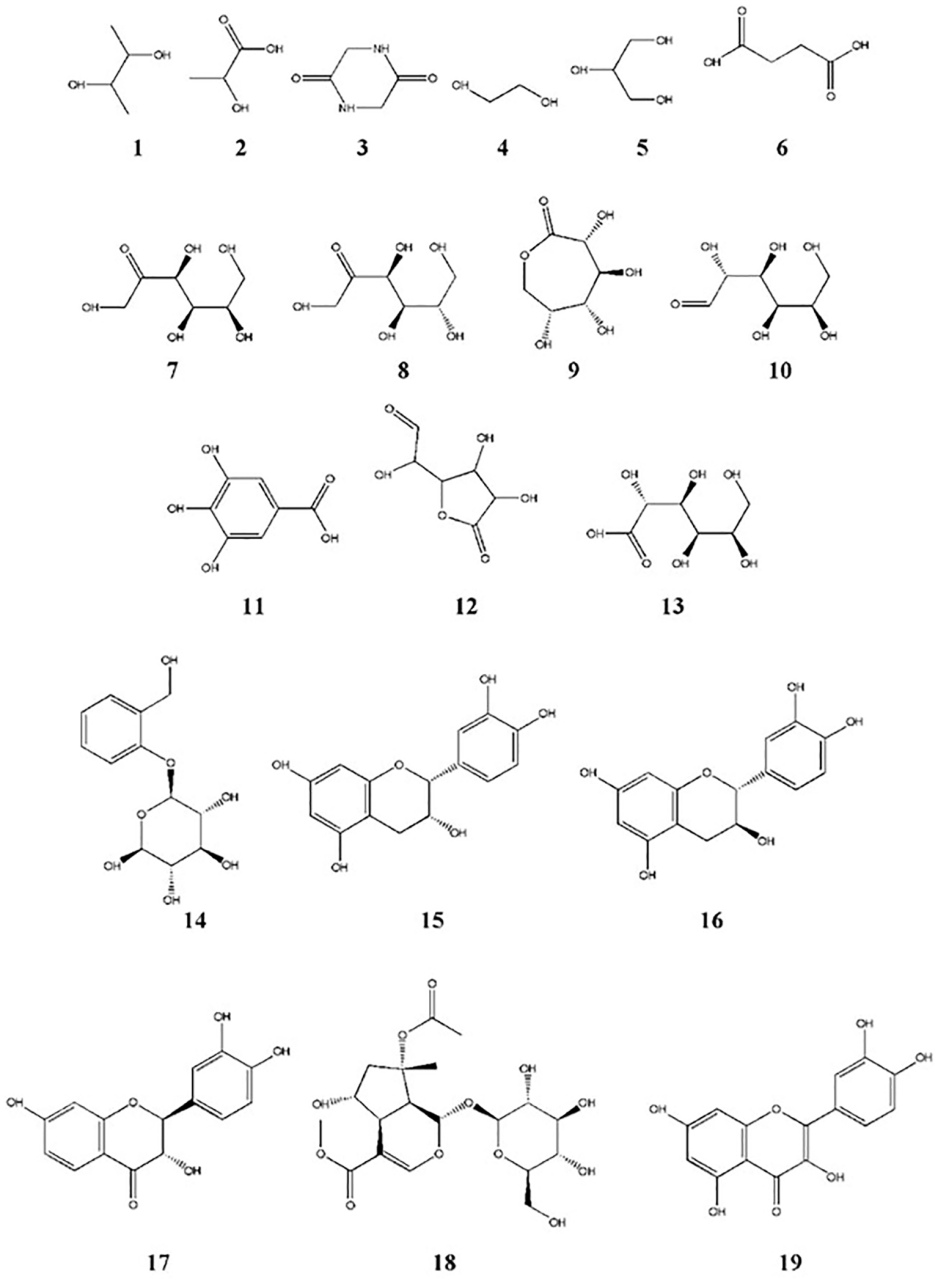
Chemical structures of compounds identified in GSE by GC-MS. Structure numbers correspond to those listed in **Table 1**.

The total phenolic content (TPC) and total flavonoid content (TFC) of GSE were 105.5 mg ± 2.81 mg of gallic acid equivalent/g dry material and 252.1 mg ± 13.63 mg of rutin equivalent/g dry material, respectively. The silylation method was followed by the GC-MS identification of 19 chemicals (**Figures 2**, **3**, **Table 1**). As shown in **Table 1**, sugars represented the most abundant group, accounting for 73.05%, while glycol structures were the least abundant at 4.55%. The percentages of organic compounds, phenolics, and flavonoids were 5.68%, 6.78%, and 9.94%, respectively.

**Table 1:** Identified compounds using GC-MS after silylation of GSE.

Peak	*t_R_*	Name	Formula	Area sum %	NP class
1	9.341	2,3-Butanediol, 2TMS derivative	C_10_H_26_O_2_Si_2_	1.55	Glycol
2	10.05	Lactic acid, 2TMS derivative	C_9_H_22_O_3_Si_2_	1.27	*Organic compound*
3	12.00	2,5-Piperazinedione, 2TMS derivative	C_10_H_22_N_2_O_2_Si_2_	1.22	Organic compound
4	12.61	Ethylene glycol, 2TMS derivative	C_8_H_22_O_2_Si_2_	0.29	Glycol
5	16.59	Glycerol, 3TMS derivative	C_12_H_32_O_3_Si_3_	2.71	Glycol
6	17.18	Butanedioic acid, 2TMS derivative	C_10_H_22_O_4_Si_2_	0.70	*Organic* compound
7	24.73	d-Fructose, 1,3,4,5,6-pentakis-*O*-(trimethylsilyl)-, *O*-methyloxime	C_22_H_55_NO_6_Si_5_	27.94	Sugar
8	24.83	l-(−)-Sorbose, pentakis(trimethylsilyl) ether, methyloxime (*syn*)	C_22_H_55_NO_6_Si_5_	25.93	Sugar
9	24.88	d-(+)-gluconolactone, 4TMS	C_18_H_42_O_6_Si_4_	2.97	Sugar
10	24.98	d-Glucose, 2,3,4,5,6-pentakis-*O*-(trimethylsilyl)-, *O*-methyloxyme, (1*Z*)-	C_22_H_55_NO_6_Si_5_	16.22	Sugar
11	25.39	Gallic acid, 4TMS derivative	C_19_H_38_O_5_Si_4_	4.17	Phenolic acid (phenolics)
12	25.71	d-(+)-Glucuronic acid γ-lactone, tris(trimethylsilyl) ether, methyloxime (anti)	C_16_H_35_NO_6_Si_3_	0.39	*Organic* compound
13	25.97	d-Gluconic acid, 6TMS	C_24_H_60_O_7_Si_6_	2.50	Organic compound
14	32.66	Salicin, 5TMS derivative	C_28_H_58_O_7_Si_5_	1.84	Phenolic glycoside (Phenolics)
15	32.80	Catechin, (2R-*cis*)-, 5TMS derivative	C_30_H_54_O_6_Si_5_	4.80	Flavanol (Flavonoids)
16	32.98	Catechin (2R-*trans*)-, 5TMS derivative	C_30_H_54_O_6_Si_5_	2.95	Flavanol (flavonoids)
17	33.45	(*S*,*S*)-3,3′,4′,7-Tetrahydroxyflavanone, 4TMS derivative	C_27_H_44_O_6_Si_4_	0.37	Flavanone (flavonoids)
18	36.99	Umbroside, 5TMS	C_34_H_68_O_12_Si_5_	0.38	Iridoid glucoside (phenolics)
19	42.31	Quercetin, 5TMS	C_30_H_50_O_7_Si_5_	1.83	Flavonol (flavonoids)
Sum				100%	

TMS, tetramethylsilane; NP, natural products.

**Figure 4:**
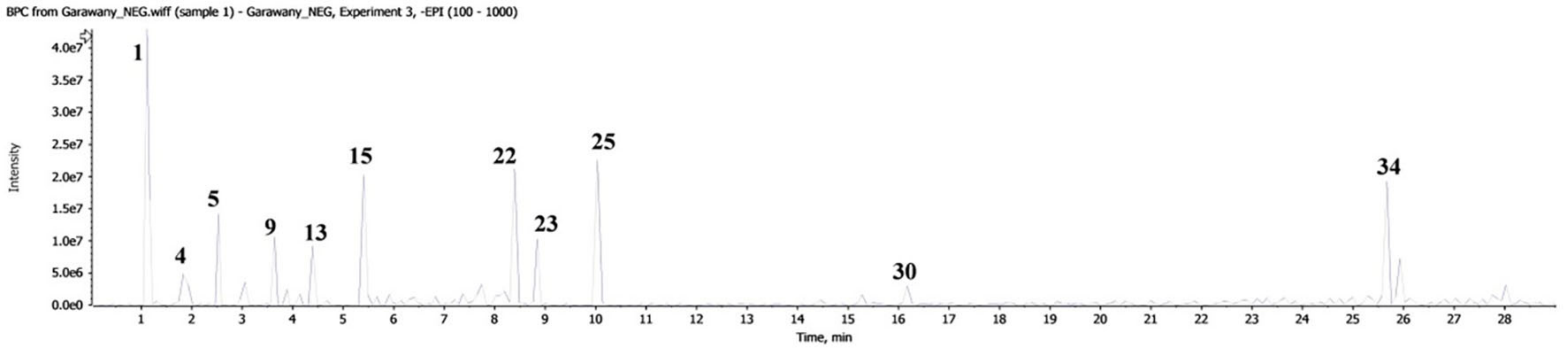
Base peak chromatogram of GSE obtained by LC-MS.

Liquid chromatography coupled with mass spectrometry (**Figure 4**, **Table 2**) revealed the presence of various phenolic structures as major secondary metabolites, along with a few fatty acid and glycerolipid structures. Among the phenolics, phenolic acids such as caffeic acid, gallic acid, *trans*-ferulic acid, and *p*-coumaric acid were characterized by the loss of 44 Daltons of CO_2_ moiety. In addition, chlorogenic acid was identified by a fragment peak at *m/z* 191, corresponding to the quinic moiety. Flavan-3-ol structures—including aglycones, dimers, trimers, and their mono- and diglycosides—were characteristic components of GSE. Flavonol structures were represented by quercetin glycosides and kaempferol aglycone. Glycosylated seco-iridoids, such as oleuropein and ligstroside, were also observed, with precursor masses of *m/z* 539 and 523, respectively. In addition, different structures of phenolics—such as dihydrochalcone, stilbenoid, flavone, dihydroxyflavone, and methylated flavone—were tentatively identified based on their molecular masses and characteristic fragment ions.

**Table 2:** Tentatively identified compounds in GSE using LC-MS/MS.

No.	*t_R_*	[M−H]^−^	Metabolites	Fragment ions (*m/z*)	NP class	Ref.
1	1.16	191	Quinic acid	147, 111	Cyclic poly Ol	Karageçili et al. (52)
2	1.31	169	Gallic acid	125	Phenolic acid	Pozzo et al. (53)
3	1.49	179	Caffeic acid	135	Phenolic acid	Pozzo et al. (53)
4	1.84	163	*p*-Coumaric acid	119	Phenolic acid	Pozzo et al. (53)
5	2.53	577	(*E*) Catechin dimer	451, 289	Flavan-3-ol	Zerbib et al. (54)
6	2.95	451	(*E*) Catechin monoglycoside	289	Flavan-3-ol-glucoside	Zerbib et al. (54)
7	3.05	577	(*E*) Catechin dimer	451, 289	Procyanidin	Pozzo et al. (53)
8	3.25	451	(*E*) Catechin monoglycoside	289	Flavan-3-ol-glucoside	Zerbib et al. (54)
9	3.42	243	Piceatannol	227	Stilbenoid	Püssa et al. (55)
10	3.90	183	Methyl gallate	125, 153, 139	Phenolic compound	Emam et al. (56)
11	4.14	183	Methyl gallate	125, 153, 139	Phenolic compound	Emam et al. (56)
12	4.36	577	(*E*) Catechin dimer	451, 289	Procyanidin	Pozzo et al. (53)
13	4.43	613	(*E*) Catechin diglycoside	577, 289	Flavan-3-ol-glucoside	Zerbib et al. (54)
14	4.50	451	(*E*) Catechin monoglycoside	289	Flavan-3-ol-glucoside	Zerbib et al. (54)
15	5.41	289	(*E*) Catechin	245, 227, 203	Flavan-3-ol	Zerbib et al. (54)
16	5.66	289	(*E*) Catechin	245, 227, 203	Flavan-3-ol	Zerbib et al. (54)
17	6.15	865	(*E*)Cat–(*E*)Cat–(*E*)Cat	847, 821, 755, 739, 712, 647, 627, 617, 577, 449, 404, 381, 327, 287	Procyanidin	Hamed et al. (57)
18	6.31	463	Quercetin-3-*O*-glucoside	301	Flavonol	Pozzo et al. (53)
19	6.41	865	(*E*)Cat–(*E*)Cat–(*E*)Cat	847, 821, 755, 739, 712, 647, 627, 617, 577, 449, 404, 381, 327, 287	Procyanidin	Hamed et al. (57)
20	7.05	539	Oleuropein	4.0, 377, 387	Secoiridoid	Pozzo et al. (53)
21	8.02	523	Ligstroside	361	Secoiridoid	Pozzo et al. (53)
22	8.58	435	Phloridzin	273	Dihydrochalcone	Pozzo et al. (53)
23	8.97	193	*trans*-Ferulic acid	149, 134	Phenolic acid	Pozzo et al. (53)
24	9.13	447	Quercetin-*O*-rhamnoside	301, 285	Flavonol	Becker et al. (58)
25	10.03	293	ND	235, 236, 231, 221, 205, 192, 177, 162, 148, 134		
26	13.38	285	Kaempferol	239	Flavonol	Karageçili et al. (52)
27	14.45	311	Arachidic acid	183, 197, 239, 225	Fatty acid	Della Corte et al. (59)
28	15.22	353	Chlorogenic acid	191	Phenolic acid	Karageçili et al. (52)
29	15.27	325	Heneicosanoic acid	183, 197, 239, 225, 281	Fatty acid	
30	16.16	339	Behenic acid	183, 197, 239, 225, 275	Fatty acid	Della Corte et al. (59)
31	18.34	227	Resveratrol	185, 143, 121, 109	Stilbenoid	Aouey et al. (60)
32	20.00	253	Chrysin	145, 119	Dihydroxyflavone	Karageçili et al. (52)
33	22.51	799	Glyceryl tripalmitoleate	255, 279	Glycerolipids	Della Corte et al. (59)
34	25.63	825	Glyceryl tripalmitoleate derivative	799, 515, 279, 255	Glycerolipids	Della Corte et al. (59)
35	25.93	269	Apigenin	151, 149	Favone	Karageçili et al. (52)
36	27.72	283	Acacetin	239	Methylated flavone	Karageçili et al. (52)

*t_R_*, retention time; *[M−H]^−^*, precursor mass; *Cat*, catechin.

### 3.2 Grape seed extract and/or ascorbic acid reduced tumor size

**Figure 5:**
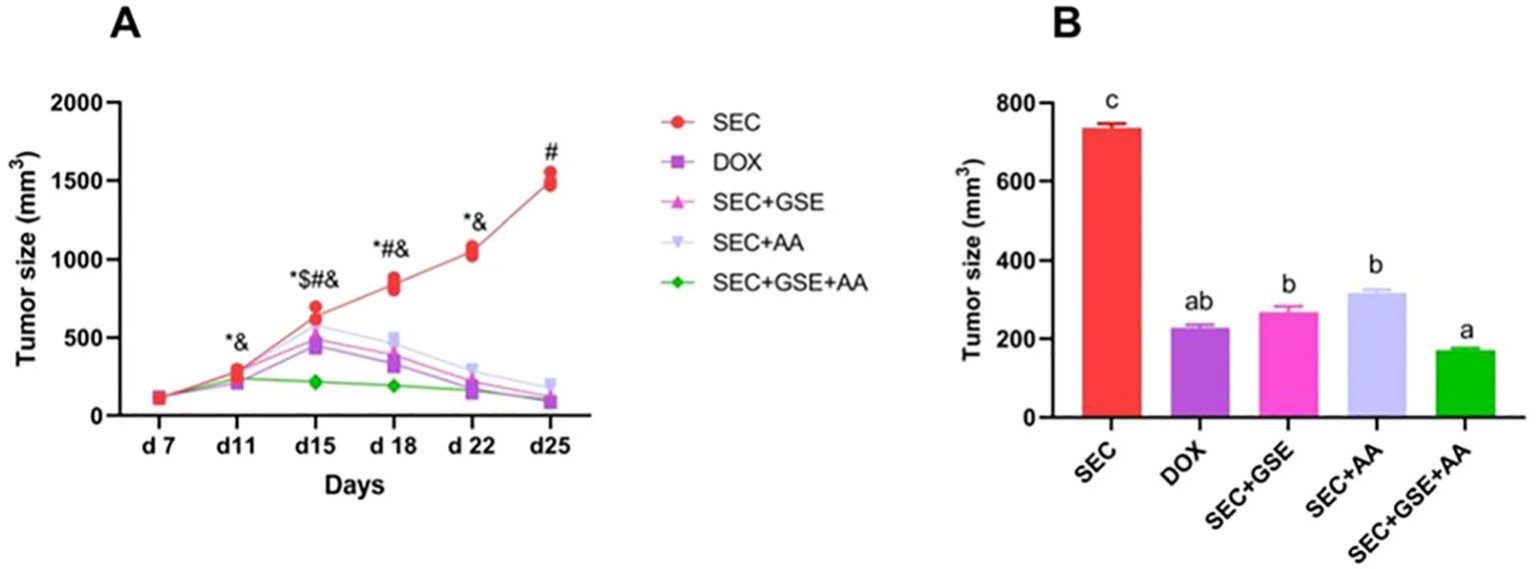
Effect of single and dual treatments with GSE and AA on tumor growth in SEC-bearing mice. **(A)** Tumor size was measured from days 7 to 25. **(B)** Final tumor size on day 25. Data are presented as mean ± SD (*n* = 5). Significant differences are indicated by an asterisk (^*^) compared to day 7, a dollar sign (^$^) compared to day 11, a number sign (^#^) compared to day 22, and an ampersand (^&^) compared to day 25 (*p* < 0.05). Different letters indicated importance (*p* < 0.05). SEC, solid Ehrlich carcinoma; DOX, doxorubicin; GSE, grape seed extract; AA, ascorbic acid.

SEC-bearing mice that received GSE and AA as single or dual treatments exhibited a significant (*p* < 0.05) shrinkage in tumor size (**Figure 5**), accompanied by a marked decrease in mean tumor volume. Tumor growth reduction reached 63.40% (mean tumor volume to 269.14 mm^3^ ± 13.69 mm^3^) in SEC-bearing mice mono-treated with GSE and 56.94% in SEC-bearing mice treated with vitamin C only (316.69 mm^3^ ± 8.74 mm^3^). Dual treatment in tumor-bearing mice with GSE plus AA resulted in a 76.61% reduction (171.977 mm^3^ ± 4.151 mm^3^), which was comparable to the 68.82% reduction in the DOX group (229.27 mm^3^ ± 6.898 mm^3^). In contrast, untreated SEC-bearing mice showed no tumor growth reduction, with a mean tumor volume of 735.40 mm³ ± 11.89 mm³.

### 3.3 Effect of the GSE and/or AA on the oxidative status in tumor tissues of mice with SEC

**Figure 6:**
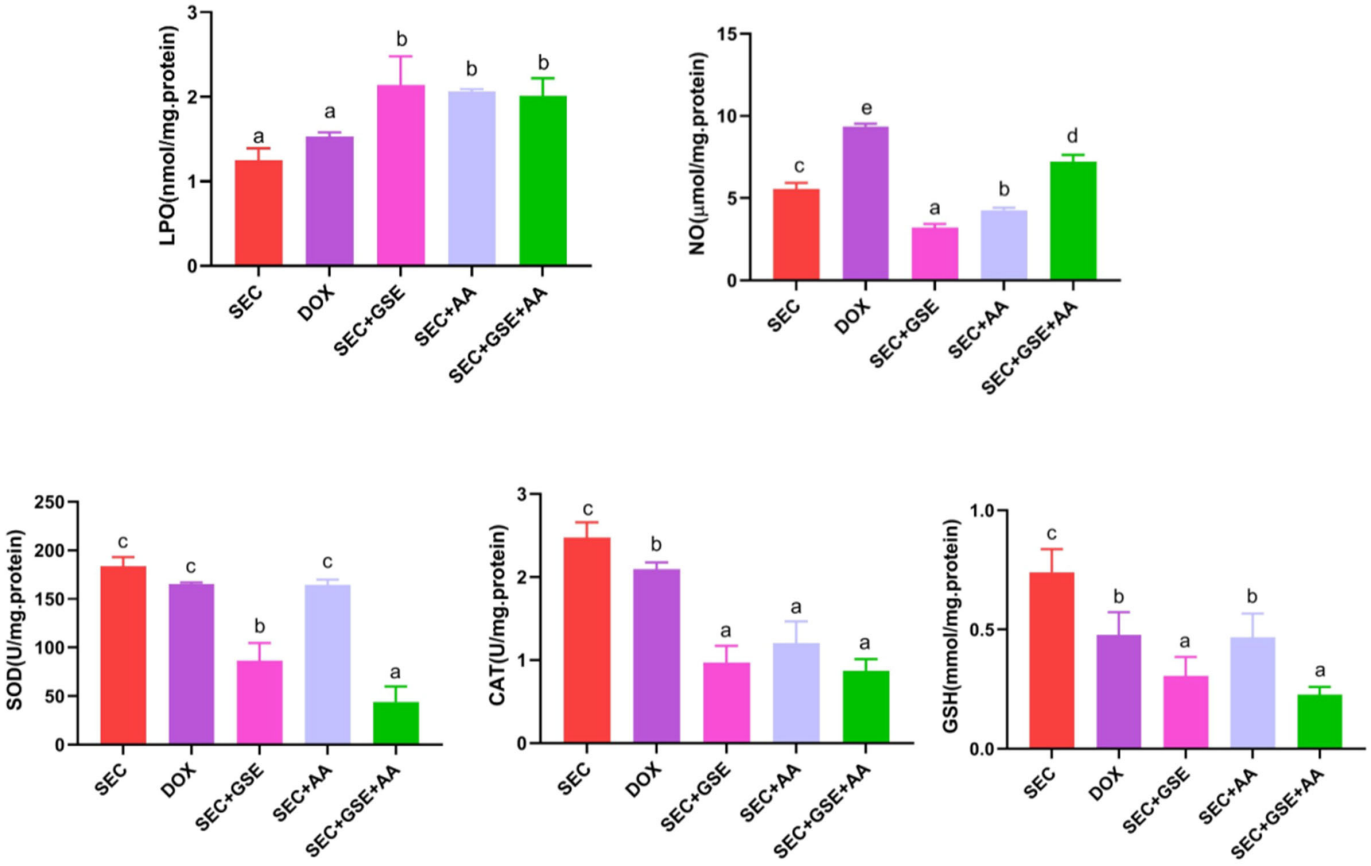
Effect of single and dual treatments with GSE and AA on tumor LPO, CAT, SOD activity, GSH, and NO concentration in SEC-bearing mice. Data are displayed as mean ± SD (*n* = 5). Different letters indicate statistically significant differences (*p* < 0.05). NO, nitric oxide; LPO, lipid peroxidase; SOD, superoxide dismutase; CAT, catalase; GSH, reduced glutathione; SEC, solid Ehrlich carcinoma; DOX, doxorubicin; GSE, grape seed extract; AA, ascorbic acid.

The effects of GSE and AA, administered as single or dual treatments, on tumor LPO, NO, SOD, CAT, and GSH levels were assessed in treated and control mice (**Figure 6**). The results showed that dual treatment with GSE and AA induced oxidative stress in solid Ehrlich tumor, as evidenced by a significant (*p* < 0.05) increase in LPO and NO by 60.8% and 29.44%, respectively. This was accompanied by a significant (*p* < 0.05) reduction in the enzymatic “CAT and SOD” by 64.899% and 79.697%, respectively, and the nonenzymatic antioxidant “GSH” by 69.29%, compared to the SEC group. Notably, monotherapy with either GSE or AA—particularly GSE—also intensified oxidative stress in tumor cells. GSE monotherapy increased LPO levels by 71.58%, while SOD, CAT, NO, and GSH levels declined by 52.85%, 60.84%, 42.322%, and 58.7%, respectively, compared to the SEC group.

### 3.4 Histopathological alterations

**Figure 7:**
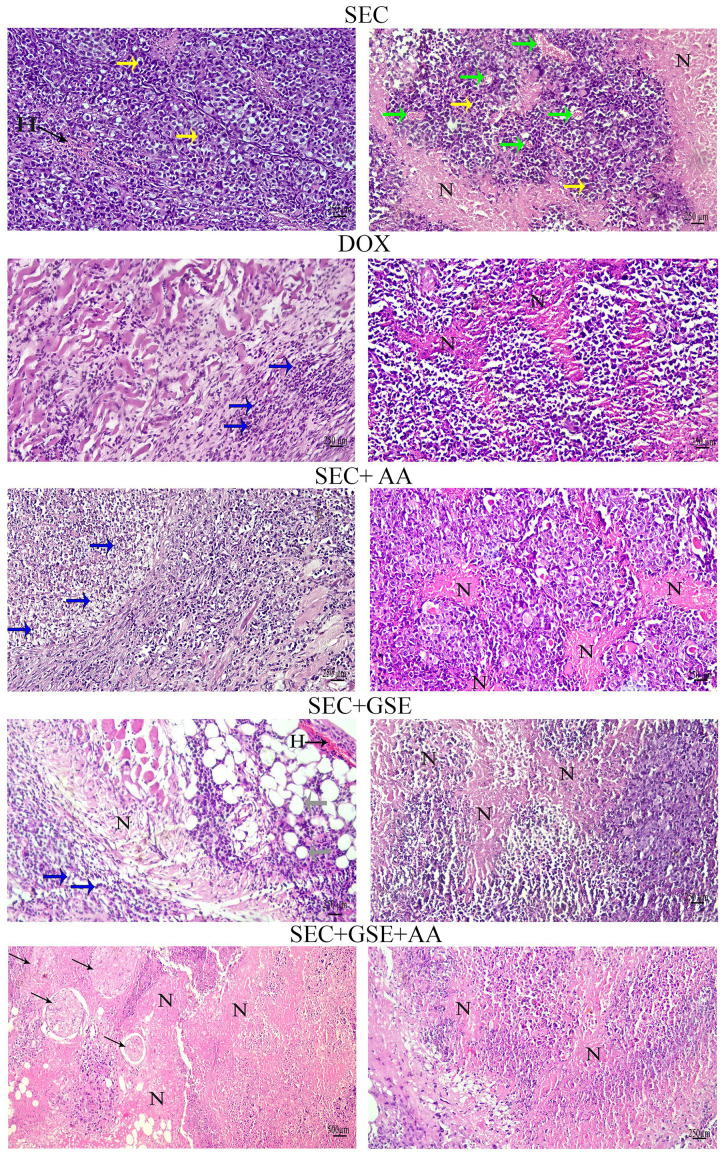
Photomicrographs of tumor sections from mice. Tumors from the SEC group showed sheets of pleomorphic, hyperchromatic malignant cells (yellow arrows), hemorrhage (H), angiogenesis (green arrows), and necrotic areas (N). Tumors from the DOX group showed necrotic areas (N) and apoptotic cells (blue arrows). Tumors from the AA group exhibited necrotic areas (N) and apoptotic cells (blue arrows). Tumor sections from the GSE group displayed extensive apoptotic cells (blue arrows), hemorrhage (H), necrotic areas (N), and adipocytes (gray arrows). The combination group (AA and GSE) showed widespread complete necrosis and cystic cells surrounded by fibers (thin arrows). (H&E stain; scale bar = 500 and 250 µm at × 10 and × 20 magnification).

Histopathological examination of the SEC group revealed numerous intramuscular masses of solid tumors in the thigh regions of mice. Tumor sections showed sheets of malignant cells with pleomorphic, hyperchromatic nuclei infiltrating between muscle bundles, necrotic areas, hemorrhage, angiogenesis, and mitotic figures. Mice treated with DOX exhibited necrotic regions and a noticeable presence of apoptotic cells within the tumor muscles. Tumors treated with ascorbic acid showed features of coagulative necrosis and apoptotic cells. In contrast, tumors exposed to GSE demonstrated extensive areas of apoptosis, hemorrhage, necrosis surrounded by fibrous tissue, and the presence of adipocytes. The combined treatment with AA and GSE demonstrated a potent antitumor effect against solid tumors, characterized by widespread areas of complete necrosis, residual tumor cells, cystic structures surrounded by fibrous tissue, and a markedly reduced number of viable tumor cells (**Figure 7**, **Table 3**).

**Table 3:** Histopathological characteristics of tumors of all groups.

Groups	Pathological features					
	Malignant cells	Necrotic areas	Angiogenesis	Apoptotic cells	Hemorrhage	Mitotic figures
SEC	+++	+	+++	+	+++	+++
DOX	+++	++	+	++	++	++
SEC + GSE	++	++	+	+++	−	−
SEC + AA	++	++	+	+++	−	−
SEC + GSE + AA	+	+++	+	+++	−	−

“−”, none; “+”, low; “++”, moderate; “+++”, high.

### 3.5 Grape seed extract and/or ascorbic acid induced tumor cell apoptosis

**Figure 8:**
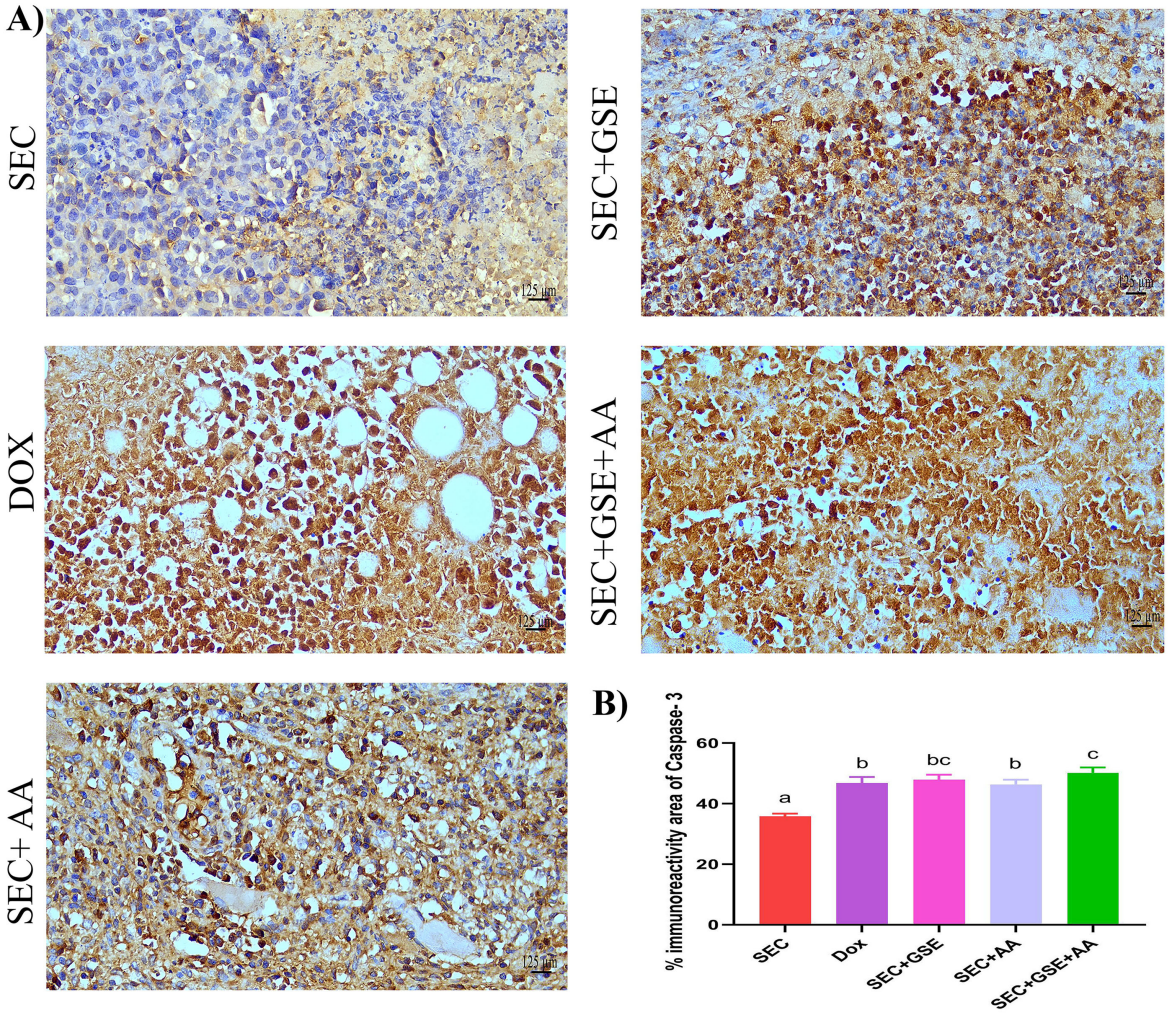
Effect of single and dual treatments with GSE and AA on tumor cell apoptosis in SEC-bearing mice. **(A)** Representative images of caspase-3 immunoreactivity in tumor tissue. **(B)** Percentage of caspase-3-positive cells across several experimental groups. Bar graphs represent the mean of six readings, expressed as mean ± SD (*n* = 5). Different letters indicate statistically significant differences (*p* < 0.05). Scale bar = 125 µm at × 40 magnification. SEC, solid Ehrlich carcinoma; DOX, doxorubicin; GSE, grape seed extract; AA, ascorbic acid.

**Figure 9:**
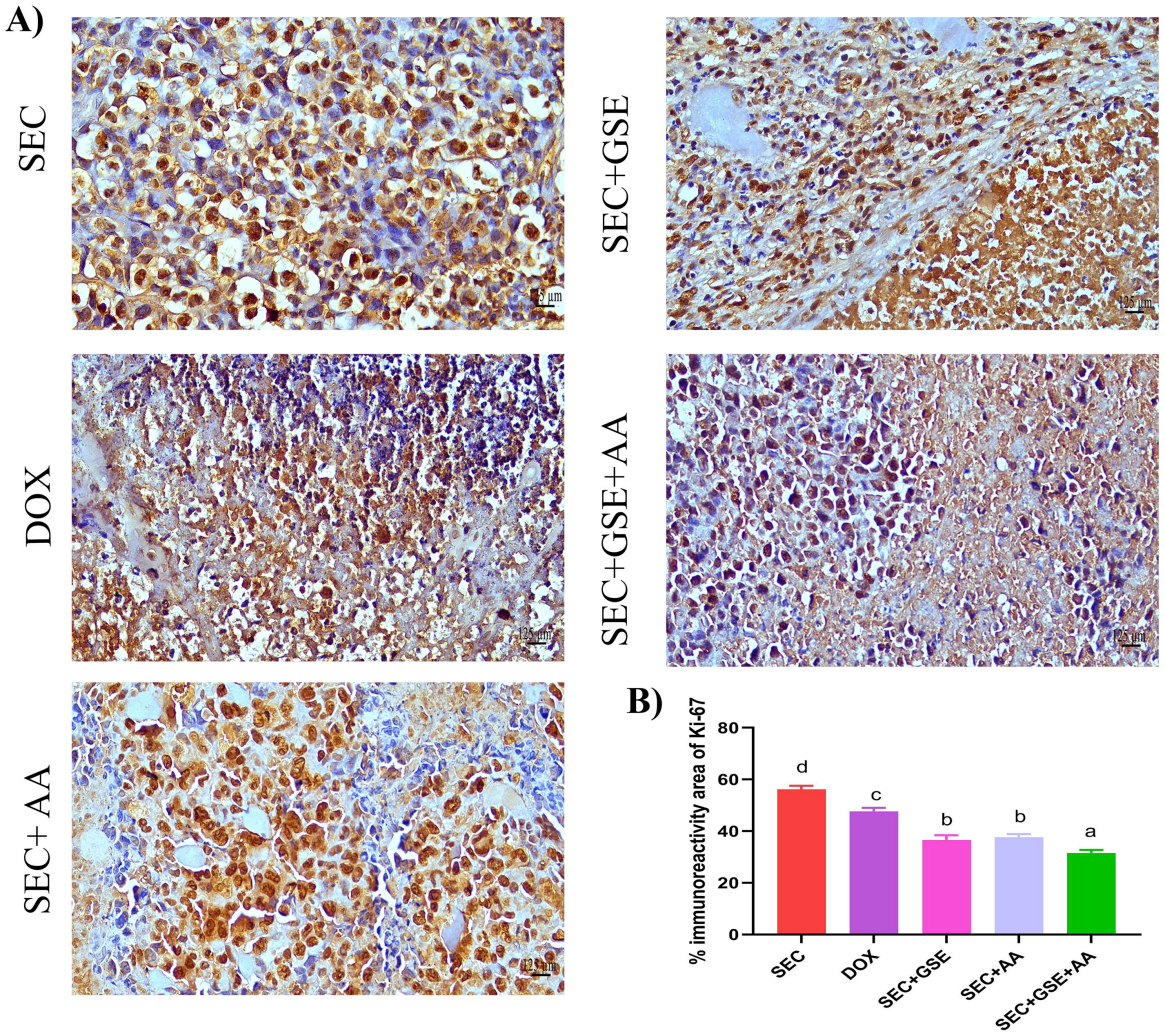
Effect of single and dual treatments with GSE and AA on tumor cell proliferation in SEC-bearing mice. **(A)** Representative Ki-67 immunoreactivity in tumor tissue. **(B)** Proportion of Ki-67-positive cells across several experimental groups. Bars represent the mean of six readings, expressed as mean ± SD (*n* = 5). Different letters indicate statistically significant differences (*p* < 0.05). Scale bar = 125 µm at × 40 magnification. SEC, solid Ehrlich carcinoma; DOX, doxorubicin; GSE, grape seed extract; AA, ascorbic acid.

Caspase-3 is a critical mediator of apoptosis, activated in the cytoplasm of apoptotic cells via both intrinsic and extrinsic pathways. Herein, caspase-3 expression levels in solid Ehrlich tumor tissues were assessed using IHC staining (**Figure 8A**). The results showed that single treatment with either GSE or AA significantly (*p* < 0.05) increased caspase-3 expression by 33.6% and 29.31%, respectively, compared to the SEC group (**Figure 9B**). Furthermore, dual treatment with GSE and AA resulted in a significant (*p* < 0.05) elevation in caspase-3 expression by 39.93% and 7.18% relative to the SEC and DOX groups, respectively (**Figure 8B**).

### 3.6 Grape seed extract and ascorbic acid dampened tumor cells’ proliferative capabilities

Ki-67 is a nuclear proliferating marker, and its overexpression is associated with poor prognosis in solid tumors. In this study, Ki-67 expression in tumor tissues was detected using IHC staining (**Figure 9A**). The results revealed that the highest Ki-67 immunoreactivity was observed in the tumor tissues of mice in the SEC group. Single treatment of SEC-bearing mice with either GSE or AA significantly (*p* < 0.05) reduced Ki-67 expression by 34.86% and 32.96%, respectively, compared to the SEC group, and by 23.21% and 20.97% compared to the DOX group. Moreover, dual treatment with GSE plus AA led to a significant (*p* < 0.05) reduction in Ki-67 immunoreactivity by 43.8% and 33.75%, compared to the SEC and DOX groups, respectively (**Figure 9B**).

### 3.7 Grape seed extract and vitamin C modulated the immune cell infiltration in tumors

**Figure 10:**
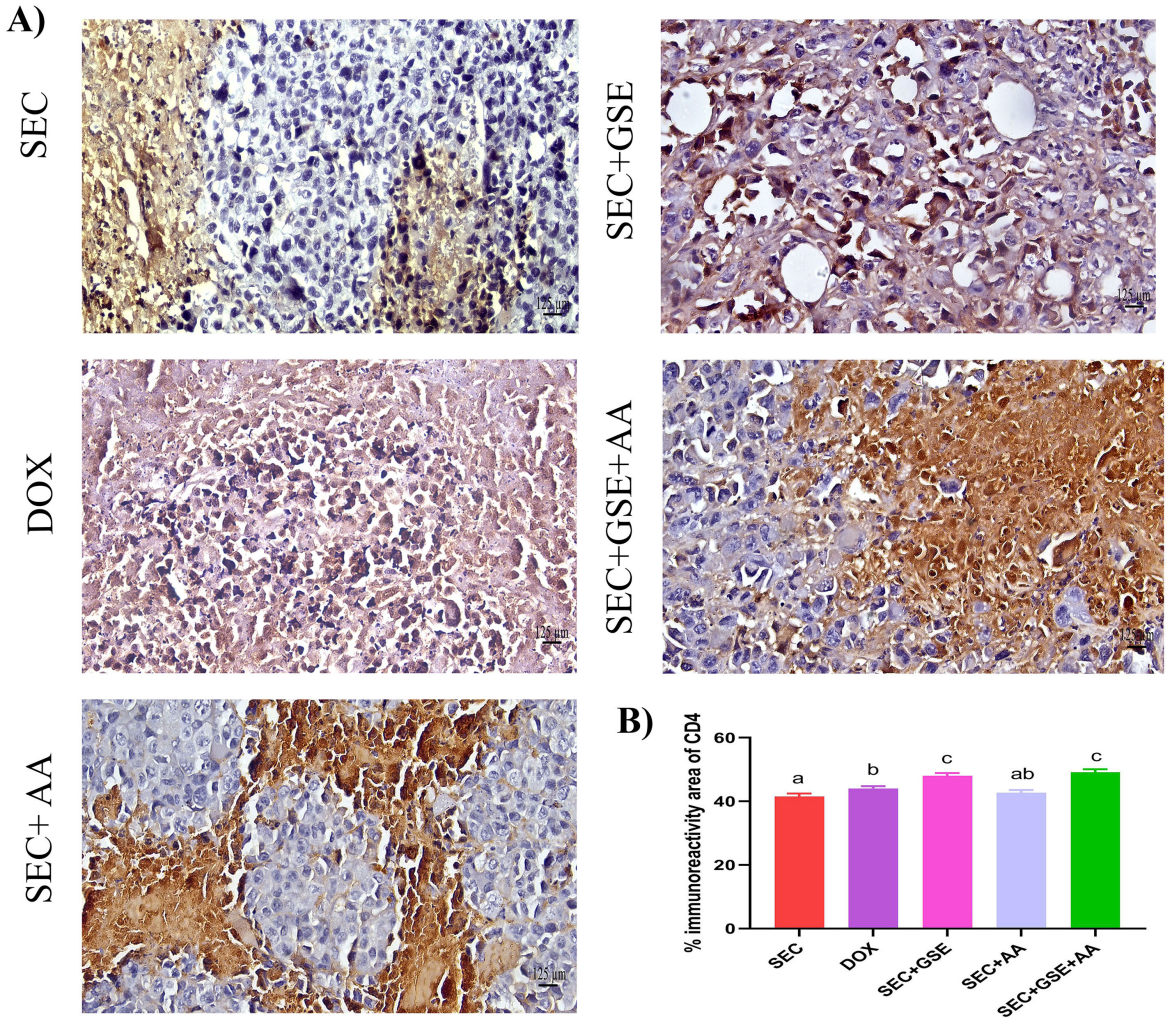
Effect of single and dual treatments with GSE and AA on infiltrated CD4^+^ cells in tumors of SEC-bearing mice. **(A)** Representative CD4^+^ immunoreactivity in tumor sections. **(B)** Percentage of CD4^+^-positive cells across several experimental groups. Bars represent the mean of six readings, expressed as mean ± SD (*n* = 5). Different letters indicate statistically significant differences (*p* < 0.05). Scale bar = 125 µm at × 40 magnification. SEC, solid Ehrlich carcinoma; DOX, doxorubicin; GSE, grape seed extract; AA, ascorbic acid.

**Figure 11:**
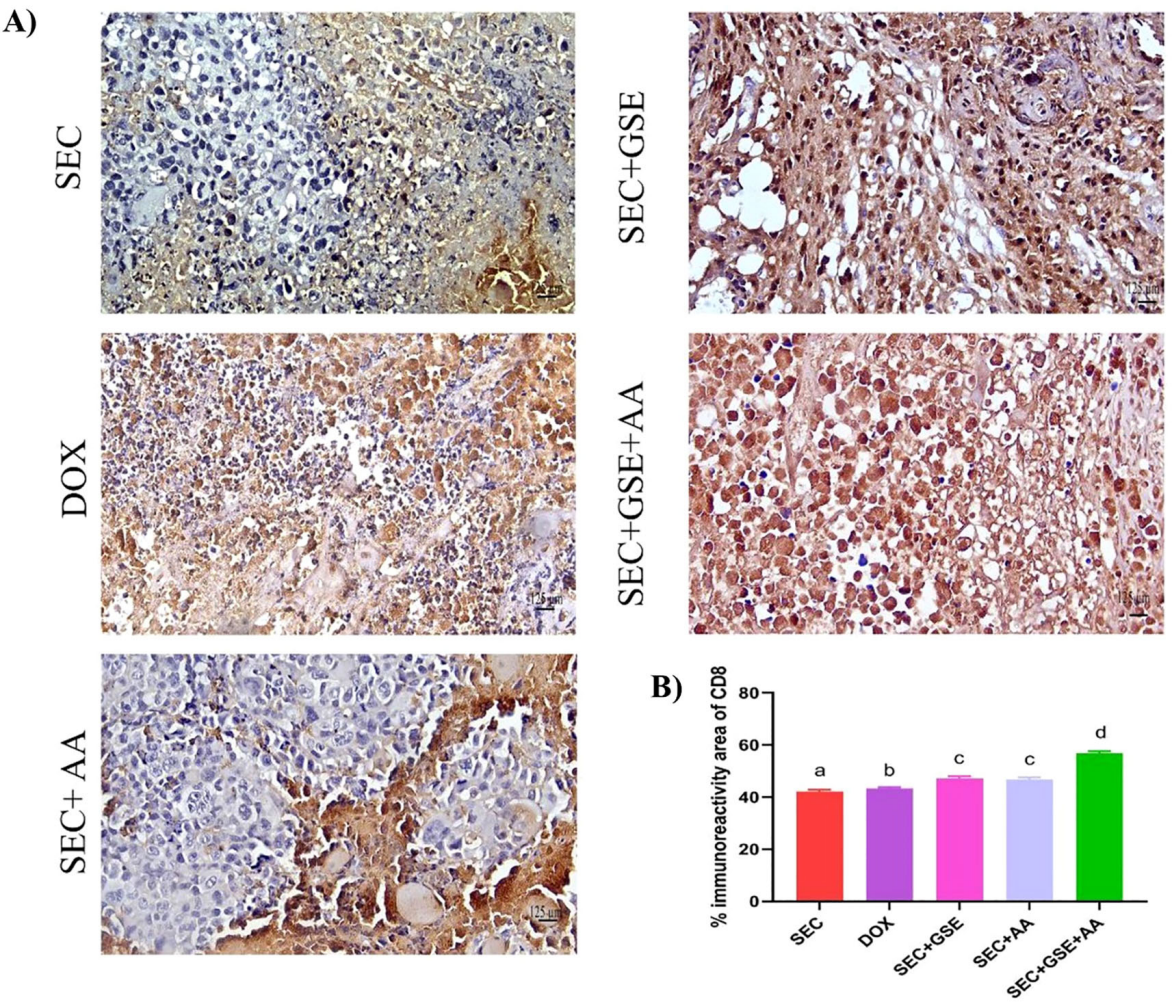
Effect of single and dual treatments with GSE and AA on infiltrated CD8^+^ cells in tumors of SEC-bearing mice. **(A)** Representative CD8^+^ immunoreactivity in tumor sections. **(B)** Percentage of CD8^+^ cells across the experimental groups. Bar graphs represent the mean of six readings, expressed as mean ± SD, with a sample size of *n* = 5. Different letters indicate significances (*p* < 0.05). Scale bar = 125 µm at × 40 magnification. SEC, solid Ehrlich carcinoma; DOX, doxorubicin; GSE, grape seed extract; AA, ascorbic acid.

**Figure 12:**
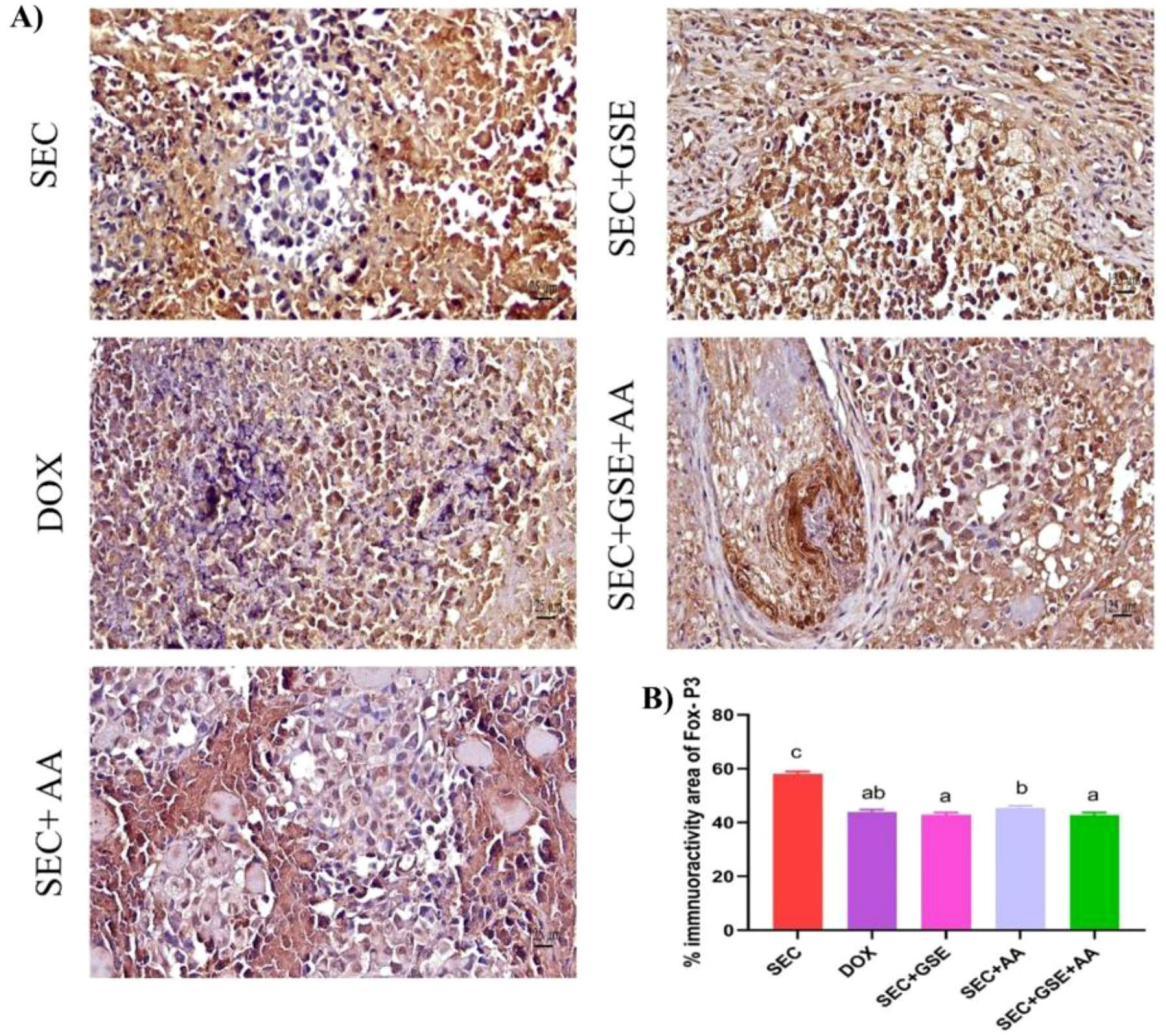
Effect of single and dual treatments with GSE and AA on infiltrated FOXP3^+^ cells in tumors of SEC-bearing mice. **(A)** Representative FOXP3 immunoreactivity in tumor sections. **(B)** Percentage of FOXP3^+^-positive cells across several experimental groups. Bars represent the mean of six readings, expressed as mean ± SD (*n* = 5). Different letters indicate statistically significant differences (*p* < 0.05). Scale bar = 125 µm at × 40 magnification. SEC, solid Ehrlich carcinoma; DOX, doxorubicin; GSE, grape seed extract; AA, ascorbic acid.

The immunoreactivity of various lymphocyte subsets infiltrating the tumors showed membranous, cytoplasmic, and peritumor staining for FOXP3, CD4, and CD8 surface markers. CD4^+^ staining was primarily observed on the membranes of helper T lymphocytes (**Figure 10A**). The results demonstrated that the number of infiltrated CD4^+^ cells in tumor tissue was significantly increased (*p* < 0.05) following treatment with GSE, showing a 15.79% rise compared to the SEC group (**Figure 10B**). CD8^+^ staining was predominantly located on the membranes of cytotoxic T lymphocytes (**Figure 11A**). Similar to CD4^+^ cells, intratumoral CD8^+^ cell infiltration significantly increased (*p* < 0.05) after the treatment with GSE and AA by 11.8% and 10.89%, respectively, compared to tumor sections from the SEC group (**Figure 11B**). FOXP3^+^ staining was primarily localized in the nuclei of regulatory T lymphocytes (**Figure 12A**). The results indicated that the number of intratumoral FOXP3^+^ Treg cells was significantly (*p* < 0.05) decreased following treatment with GSE and AA by 26.29% and 21.86%, respectively, compared to tumor sections from SEC-bearing mice (**Figure 12B**). Overall, cotreatment with GSE and AA enhanced the immune competence of the tumor microenvironment. Compared to the SEC group, the combined treatment significantly increased CD4^+^ and CD8^+^ cell infiltration by 18.45% and 34.85%, respectively, while significantly decreasing FOXP3^+^ cell expression by 26.43%.

### 3.8 Grape seed extract and/or ascorbic acid boosted Th1/Th2 into a Th1 response

**Figure 13:**
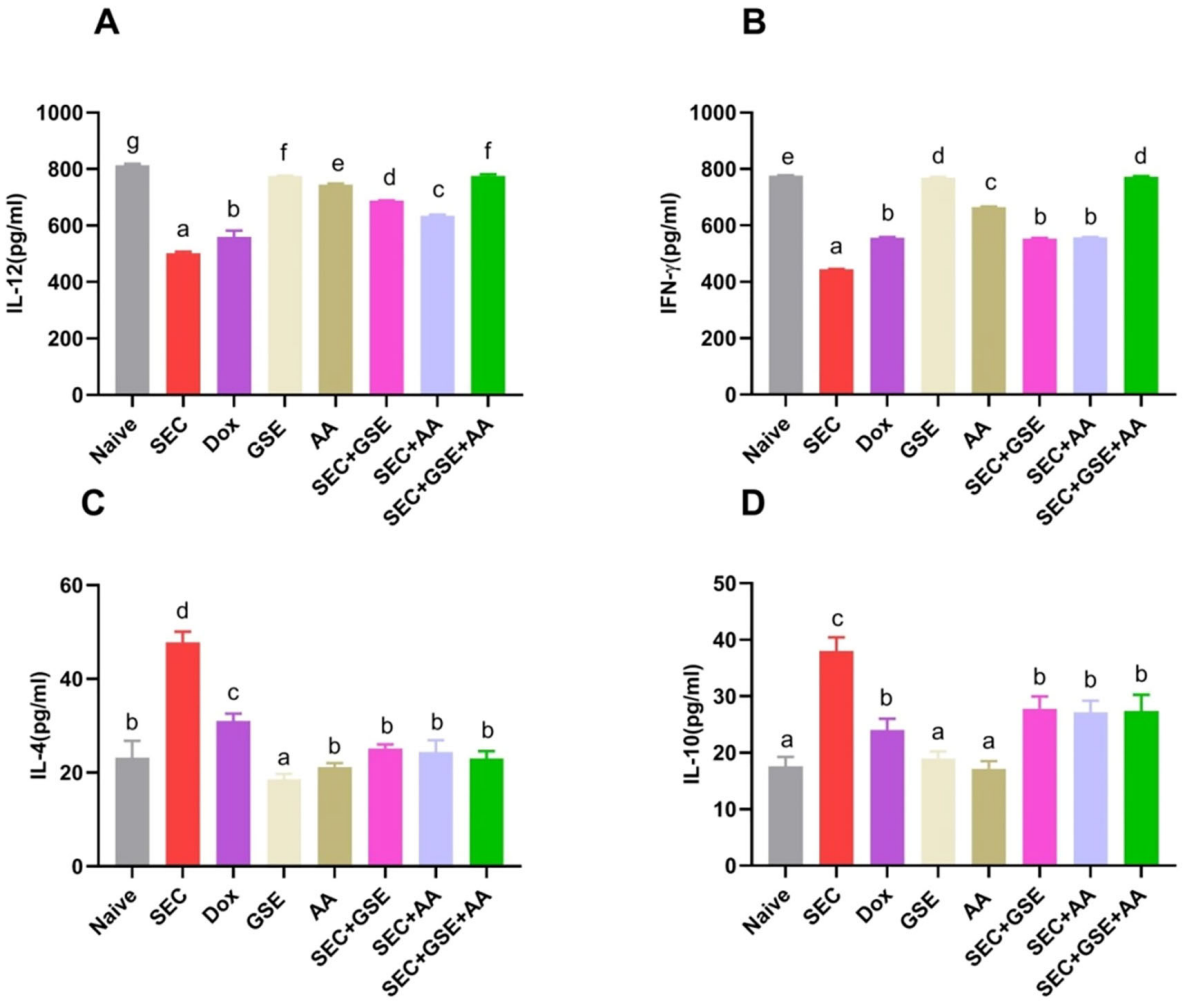
Effect of single and dual treatments with GSE and AA on Th1/Th2 cytokines in SEC-bearing mice. **(A, B)** Serum levels of Th1 cytokines IL-12 and IFN-γ; **(C, D)** Serum levels of Th2 cytokines IL-4 and IL-10. Data are presented as mean ± SD (*n* = 5). Different letters indicate statistically significant differences (*p* < 0.05). SEC, solid Ehrlich carcinoma; DOX, doxorubicin; GSE, grape seed extract; AA, ascorbic acid.

The effects of GSE and/or AA on the T-cell-mediated antitumor immune response were evaluated by measuring Th1 (IFN-γ and IL-12) and Th2 (IL-4 and IL-10) cytokines, as shown in **Figure 13**. IL-4 and IL-10 levels were significantly decreased in SEC-bearing mice treated with GSE, AA, and or their combination compared to untreated SEC-bearing mice. Specifically, IL-4 levels were 25.2 ± 0.84, 24.4 ± 2.5, and 23 ± 1.58 in mice treated with GSE, AA, or both, respectively, compared to 47.8 ± 2.28 in the untreated SEC-bearing mice. Similarly, IL-10 levels were 27.8 ± 2.17, 11.14 ± 2.05, and 27.4 ± 2.88 in the treated groups, versus 38 ± 2.45 in the untreated SEC-bearing mice. Additionally, increased levels of IL-12 and IFN-γ were observed in all SEC-bearing mice treated with GSE and AA, either as single or dual treatments, compared to untreated SEC-bearing mice. The DOX group showed a significant decrease in IL-4 (31 ± 1.58) and IL-10 (24 ± 2) levels compared to the untreated SEC-bearing mice. Additionally, IL-12 and IFN-γ levels in the DOX group significantly increased to 559.6 ± 22.86 and 556 ± 2.45, respectively, compared to 501.6 ± 5.27 and 443.6 ± 1.82 in the untreated SEC-bearing mice. Notably, the highest and most adverse IL-12 and IFN-γ levels were observed in the dual-treatment group, exceeding those of all other groups. The results indicated that both single and dual treatments with GSE and AA promoted the release of IL-12 and IFN-γ while suppressing the secretion of IL-10 and IL-4. These findings suggest that GSE and AA may shift the Th1/Th2 balance toward a Th1 cell-mediated immune response.

## 4 Discussion

Cancer eradication remains one of the most challenging global medical issues, highlighting the urgent need for new alternatives to supplement the limited supply of antitumoral drugs. This study aimed to explore the potential antitumor and immunomodulatory effects of a dual treatment using red grape seed extract combined with vitamin C in a murine model of solid Ehrlich carcinoma.

GC-MS and LC-MS techniques were used to determine the chemical profile of the GSE extract used in the study. This profile was established based on the spectral fragmentation patterns, molecular masses identified, and retention times observed in each chromatogram, allowing compound identification through comparison with literature data. The major compounds detected included gallic acid, catechins, quercetin, caffeic acid, kaempferol, resveratrol, and apigenin—compounds previously reported to possess antioxidant, anti-inflammatory, and antineoplastic propoerties (29, 61–73). The chemical structure of grape seed compounds can be directly linked to their tumor-inhibitory and immune-regulatory effects. Moreover, the micronutrient vitamin C (l-ascorbic acid), essential for numerous physiological functions, has demonstrated cytotoxic against various cancer types. Despite its general nontoxicity, its potential role in cancer prevention and treatment underscores its promise as a lead compound for novel anticancer therapies (74). To the best of our knowledge, this study is the first to evaluate the anticancer efficacyof ascorbic acid, grape seed extract, or their combination in tumor-bearing mice, and to investigate their impact on the tumor immune microenvironment.

In this study, GSE and AA cotreatment showed antineoplastic potencies in SEC-bearing mice by inhibiting cancer growth. A similar pattern was observed in SEC-bearing mice receiving GSE or AA as monotherapy, with no significant difference compared to DOX-treated animals from day 11 until the end of the experiment. The antineoplastic properties of GSE and AA have been previously reported (75–81).

Cancer cells are particularly susceptible to oxidative injury due to their elevated basal levels of ROS (82). Moreover, increased NO levels can be cytotoxic to cancer cells by generating peroxynitrite, a potent inducer of apoptosis and other damaging species involved in immune surveillance (83). In our study, dual treatment with GSE and AA intensified oxidative stress in Ehrlich carcinoma cells by upregulating NO levels and initiating lipid peroxidation, as evidenced by increased LPO levels. This was accompanied by a reduction in GSH content and decreased activities of SOD and CAT. A similar effect was observed with a single treatment using either GSE or AA, with GSE showing greater potency. These outcomes matched those of Shrotriya et al. (75), who reported that GSE caused accumulation of intracellular ROS in HNSCC, which was behind its tumor growth inhibition, DNA destruction, and cell death, and this was dramatically mitigated by *N*-acetylcysteine. Accordingly, GSE administration was previously evidenced to induce considerable superoxide radical-linked oxidative stress and a large drop in intracellular GSH levels. This suggests that GSE-induced oxidative stress plays a role in its ability to trigger apoptosis against non-small cell lung cancer (84). In context, vitamin C exerts a lethal effect on cancer cells by enhancing oxidative stress via two primary mechanisms: the production of DHA and Fe^2+^ during its oxidation within the tumor microenvironment (85, 86). Increased levels of labile ferric iron (Fe^3+^) in the tumor microenvironment can facilitate vitamin C oxidation, resulting in the generation of DHA and ferrous iron (Fe^2+^). H_2_O_2_ reacts with the generated Fe^2+^ to produce highly reactive hydroxyl radicals (·OH), which can cause direct cell membrane damage via lipid peroxidation (87).

Tumor cells are primarily characterized by uncontrolled and uncontrolled proliferation and evasion of apoptotic processes (88). To determine the mechanisms underlying the antineoplastic potency of GSE- and/or AA-based therapy, SEC-bearing mice were monitored by assessing cancer cell proliferative capacity and apoptotic profile using Ki-67 and caspase-3, respectively. Notably, tumorized mice that received dual treatment with GSE and AA exhibited a significant upregulation of caspase-3 expression, accompanied by a significant downregulation in Ki-67 expression, compared to those in the SEC and DOX groups. Consistently, ascorbic acid induced apoptosis in gastric cancer cells by disrupting mitochondrial function, including ATP consumption, ROS production, and calcium influx (89). Furthermore, vitamin C triggered apoptosis in both nonaggressive and aggressive MCF-7 breast cancer cell lines through oxidative stress generation (90). Similarly, the apoptotic and antiproliferative efficacies of GSE have been reported both *in vitro* and *in vivo* against various cancer types, including prostate cancer (91–93), colorectal cancer (41, 94), breast cancer (41, 95), human bladder carcinoma (96), and ovarian cancer (97).

Quercetin, a potent ingredient of GSE, has been reported to promote apoptosis by lowering the expression levels of antiapoptotic proteins and increasing the expression levels of proapoptotic proteins, while also inhibiting tumor cell proliferation and disrupting cell cycle progression in A375SM melanoma cells, A2780S ovarian cancer cells, HL-60 AML cells, and gastric cancer cells (65, 68, 98–101). In addition, gallic acid, another active constituent of GSE, has been shown to exert proapoptotic, antimigratory, and antiproliferative effects against NSCLC both *in vivo* and *in vitro* (64). It has been proposed that the antitumor effect of gallic acid is associated with autophagy. Autophagy can induce tumor cell apoptosis by degrading the endoplasmic reticulum, Golgi apparatus, and other cellular components, leading to protein imbalance in cancerous cells and thereby suppressing uncontrolled proliferation (64, 102, 103). Furthermore, Sarı et al. (104) reported the proapoptotic effect of gallic acid in HeLa cancer cells through activation of the p53/Bax signaling pathway. Another constituent of GSE under investigation is catechin, which has also been shown to exert antiproliferative and apoptotic effects against murine lymphoma cells LB02 by increasing Bax expression and downregulating Bcl-2 and survivin (105). Similarly, Dükel et al. (106) reported the apoptotic potency of catechin via upregulation of cell-death-related genes in human colon cancer cells.

Antitumor immunity relies heavily on lymphocytes (107). Improved clinical outcomes and survival in cancer patients are associated with tumor-infiltrating T cells (108, 109). T-cell subpopulations form a complex network within the tumor microenvironment, and the Th1/Th2 balance among these subgroups influences the immune response in malignancy. A shift from a Th1- to a Th2-dominant response is known to promote carcinogenesis (110). To examine how GSE and/or AA affect T cells in the tumor microenvironment, various tumor-infiltrating lymphocyte (TIL) subsets were identified. Both innate and adaptive immunity are essential components of antitumor defense. Cellular and humoral immunity are the two main arms of the acquired immune response. CD8^+^ and CD4^+^ T cells play pivotal roles in cellular immunity (111, 112). CD8^+^ cytotoxic lymphocytes mediate antitumor immunity by recognizing “foreign-looking” antigens on the surface of cancer cells (113). However, their capacity to eliminate tumor cells, secrete inflammatory mediators (such as TNF-α and IFN-γ), proliferate, and form long-term memory cells can be impaired through T-cell receptor desensitization. This desensitization is often driven by the overexpression of inhibitory receptors such as LAG-3, TIGIT, PD-1, and TIM-3 (114). Contrarily, FOXP3^+^ regulatory T cells contribute to a suppressive tumor microenvironment, facilitating tumor immune evasion (115, 116). The results revealed that dual treatment with GSE and AA considerably increased CD8^+^ and CD4^+^ T-cell infiltration, accompanied by a decrease in FOXP3^+^ Treg cells in the SEC microenvironment. Gallic acid, a constituent of GSE, was found to enhance intratumoral CD8^+^ T cells while suppressing tumor-infiltrating FOXP3^+^ Treg cells in a murine model of colorectal carcinoma (117). Notably, ascorbic acid has also been shown to modulate the tumor microenvironment by promoting T-lymphocyte infiltration (118). Moreover, Magrì et al. (119) reported that ascorbic acid can regulate immune cell infiltration into the tumor microenvironment in mice with syngeneic tumors. To further clarify the effects of GSE and/or AA on the Th1/Th2 balance, cytokines released by Th1 and Th2 cells were analyzed. The results showed that dual treatment with GSE and AA led to a remarkable increase in the Th1 cytokines IL-12 and IFN-γ, accompanied by decreased levels of Th2 cytokines IL-4 and IL-10. This indicates that dual treatment shifted the Th1/Th2 balance toward a Th1-dominant response. These findings suggest that GSE and AA inhibit tumor growth by promoting a Th1/Th2 balance in favor of the Th1 response, which is consistent with the findings of Zhao et al. (111). Accordingly, Nair et al. (120) reported that GSE stimulated the Th1 response through the induction of IFN-γ. Similarly, ascorbic acid has previously been reported to modulate Th1/Th2 balance in favor of the Th1 pole (121–125). In addition, Qin et al. (126) demonstrated that ascorbic acid enhanced Th1 immune response and dendritic cell activity in *Plasmodium yoelii* 17XL-infected mice. Notably, quercetin has also been shown to regulate the Th1/Th2 balance by upregulating Th1 cytokine levels and suppressing Th2 cytokine levels through modulation of *T-bet* and *GATA-3* gene expression in an experimental asthma model (127). Consistently, quercetin and gallic acid have been shown to promote Th1/Th2 balance and regulate Treg/Th17 ratios by activating the NF-κB pathway in allergic diseases (61, 66, 128). Interestingly, GSE and vitamin C can enhance IFN-γ production, indicating a clear shift toward a Th1 response, which activates the cellular immune compartment and facilitates cancer cell destruction through mechanisms that are independent of natural killer (NK) natural killer T (NKT) cells, but dependent on CD8^+^ T-cell recognition via MHC class I presentation (129, 130). Moreover, IFN-γ promotes a favorable polarization of intratumoral macrophages toward the M1 phenotype, enhancing their ability to eliminate cancer cells (131).

## 5 Conclusion

The presented findings demonstrate that cotreatments with GSE and/or vitamin C hold promise as an anticancer strategy by reducing tumor size, modulating the intratumoral immune response, inducing oxidative stress, decreasing tumor cell proliferation, and promoting tumor cell death. The authors recommend initiating clinical trials to evaluate human pharmacokinetics, safety, and efficacy, particularly given the use of natural compounds. A key limitation of this study is its focus on short-term tumor progression and acute treatment responses in the solid Ehrlich carcinoma model. As such, long-term therapeutic durability, including tumor recurrence and potential resistance mechanisms, could not be assessed. Future investigations with extended follow-up are necessary to fully characterize the sustained efficacy and biological impact of the treatments in this model. Although the study provides compelling evidence that grape seed extract and l-ascorbic acid exert antineoplastic effects via immunomodulation and alterations in the tumor microenvironment, particularly through modulation of the Th1/Th2 balance, this focus may oversimplify the complex immune dynamics involved in tumor progression. More comprehensive immunological and molecular investigations are needed to elucidate the full spectrum of immune mechanisms underlying these effects.

## Data Availability

The original contributions presented in the study are included in the article/supplementary material. Further inquiries can be directed to the corresponding author.
